# Analysis of Risk Factors of Gastric Low-Grade Intraepithelial Neoplasia in Asymptomatic Subjects Undergoing Physical Examination

**DOI:** 10.1155/2020/7907195

**Published:** 2020-03-03

**Authors:** Yingling Liu, Yuli Cai, Si Chen, Yawen Gou, Qiaomin Wang, Mingli Zhang, Yetao Wang, Haiou Hong, Kaiguang Zhang

**Affiliations:** ^1^Department of Gastroenterology, The First Affiliated Hospital of USTC, Division of Life Sciences and Medicine, University of Science and Technology of China (Anhui Provincial Hospital), Hefei, China; ^2^The Physical Examination Center, The First Affiliated Hospital of USTC, Division of Life Sciences and Medicine, University of Science and Technology of China (Anhui Provincial Hospital), Hefei, China

## Abstract

Secondary prevention is an important strategy in gastric cancer. Low-grade intraepithelial neoplasia (LGIN) is the last stage of precancerous lesion, and its timely diagnosis can greatly improve the detection rate of early gastric cancer. We performed a prospective study to analyze the risk factors of gastric LGIN in asymptomatic subjects undergoing physical examination. A total of 3437 subjects were included in this study, and 2259 asymptomatic subjects were investigated from March 2015 to April 2018. Risk factors were evaluated, and the endoscopic features of LGIN and prognosis were described. The overall incidence of LGIN was 19.73% (678/3437), while the incidence of LGIN in the asymptomatic and symptomatic groups was 19.65% (444/2259) and 19.86% (234/1178), respectively (*P* = 0.884). The rate of *Helicobacter pylori* infection in this physical examination population was 39.13% (35.8% asymptomatic group, 45.5% symptomatic group; *P* ≤ 0.001). Risk factors including age, *H. pylori* infection, history of antibiotic misuse, and spicy and high-fat diet (all *P* < 0.05) were further verified by multivariate analysis as independent risk factors. History of antibiotic misuse and *H. pylori* infection showed significant associations with LGIN (odds ratio (OR) = 6.767, 95% confidence interval (CI) 3.873-11.825 and OR = 3.803, 95% CI 3.009-4.808, respectively). The most common endoscopic classification of LGIN was erosive gastritis (50.78%), and the major endoscopic appearance was Paris IIa (flat with slight elevation located mostly in the antrum). During the mean follow-up period of 15.02 months, 49.4% of LGIN regressed, 0.61% of LGIN progressed, and 50% of LGIN remained unchanged. History of antibiotic misuse and *H. pylori* infection were predominant risk factors of LGIN in asymptomatic subjects, and those individuals should consider early screening for gastric cancer.

## 1. Introduction

Worldwide, gastric cancer ranks fifth in terms of malignancies and it is the third leading cause of death due to malignant tumors [1]. It is estimated that there are 423,000 new cases and 300,000 deaths in China per year, accounting for 44.5% of the global incidence of gastric cancer [2]. The majority of cases can be treated radically by endoscopy at an early stage, with a >90% 5-year survival rate [3]. However, the current diagnosis and treatment of early gastric cancer in China is <20% of all reported cases and certainly requires significant improvement to ensure more efficient treatment of gastric cancer. Because of the large population in China, endoscopy cannot be used as a general screening method for all early gastric cancers and precancerous lesions. A more realistic and cost-effective method is to identify people at high risk for developing gastric cancer prior to further clinical scrutiny. The development of gastric cancer is a multi-factorial and multi-step process, and low-grade intraepithelial neoplasia (LGIN) of the gastric mucosa represents the final stage of gastric precancerous lesion (equivalent to mild and moderate dysplasia) [4]. Therefore, identification of risk factors for LGIN would provide key insights into the prevention and treatment of gastric cancer. In this study, we focused on the asymptomatic population undergoing physical examination to identify risk factors of LGIN. Using this research cohort offers an advantage in avoiding differences in the diagnosis and treatment process of symptomatic patients.

Our analysis revealed that LGIN was associated with multiple independent risk factors that could facilitate a better diagnosis and treatment of early gastric cancer and precancerous lesions. This will provide useful information for the secondary prevention of gastric cancer.

## 2. Patients and Methods

### 2.1. Study Population and Data Collection

We prospectively selected cases of endoscopy registered in Anhui Provincial Hospital Physical Examination Center from March 2015 to April 2018. All of the participating volunteers were over 18 years old and had signed informed consent to accept endoscopy and pathological examination voluntarily as research subjects. The exclusion criteria were as follows: severe liver and kidney function impairment, anemia, coagulation dysfunction, serious cardiovascular and cerebrovascular diseases, history of gastric surgery, and tumor history.

Before the gastroscopy procedure, the purpose of this examination was explained to the subjects by filling out questionnaires, and they were divided into two groups: (1) no gastrointestinal symptoms (asymptomatic) and (2) gastrointestinal discomfort or slight physical discomfort (symptomatic). All subjects provided information on 12 items in detail: sex, age, height, weight, history of nonsteroidal anti-inflammatory drugs (NSAIDs), history of antibiotic misuse, family history of gastric cancer, smoking history, alcohol history, high-salt diet, spicy and fried diet, and poor eating habits ([Supplementary-material supplementary-material-1]). *Helicobacter pylori* infection was tested by ^13^C urea breath test.

### 2.2. Gastroscopy Procedure

All subjects were fasted more than 8 h prior to the procedure. To clean and numb the throat, subjects were asked to consume 10 ml of Xisha silicone oil emulsion with 15 ml of saline mixture before the procedure. Gastroscopy was completed by two senior endoscopy experts with >10 years of experience who made a detailed inspection of the stomach using conventional white-light endoscopes, sometimes performing narrow band imaging and magnifying endoscopy if necessary. Gastric biopsy sampling followed the standard biopsy sampling protocol of the gastric mucosa: one from the incisura angularis; two from the large and small bend in the antrum (2-3 cm from the pylorus); and two from the large and small bend in the stomach body (8 cm from the cardia). Additional samples were always taken from any focal lesions (ulcer, mucosal flat abnormalities, and polyps). Biopsy samples were large enough to reach the mucosal muscle.

Endoscopic classification of chronic gastritis was categorized into chronic superficial gastritis, pangastritis, chronic atrophic gastritis, erosive gastritis, atrophic mixed erosive gastritis, and gastric ulcer [5]. The description of endoscopic appearances of LGIN lesions was reclassified in accordance with the Paris classification of superficial neoplastic lesions [6].

### 2.3. Pathology

The specimens were fixed in 10% formalin solution, embedded in paraffin, and stained with hematoxylin violet. The histological changes of chronic inflammation, activity, atrophy, intestinal metaplasia, and intraepithelial neoplasia were observed by pathologists according to the New Sydney Gastritis Classification [7] and Vienna Classification [8].

The resulting specimens were viewed by three designated senior pathologists, and the same diagnoses reached by two out of the three pathologists were recorded as the final diagnosis. We excluded endoscopic manifestations of other lesions, such as esophageal or duodenal lesions, or pathological findings of inflammatory polyp, high-grade intraepithelial neoplasia (HGIN), and gastric malignancies.

### 2.4. Follow-Up

Pathologically proved cases of gastric LGIN in asymptomatic patients were followed up every 6 months to 1 year, including *H. pylori* eradication, gastroscopy, and pathology.

### 2.5. Statistical Analysis

The relationships between LGIN incidence and the 12 potential risk factors were investigated by analyzing the data with SPSS 17.0 statistical software (SPSS Inc., Chicago, IL, USA). Specifically, the data were first analyzed by the chi-squared test with single-factor analysis to control confounding factors, and *P* < 0.05 was considered statistically significant. Binary logistic regression analysis of multifactor analyses was subsequently performed to explore the impact of independent risk factors on LGIN of the gastric mucosa.

## 3. Results

### 3.1. Basic Data Characteristics

**Figure 1:**
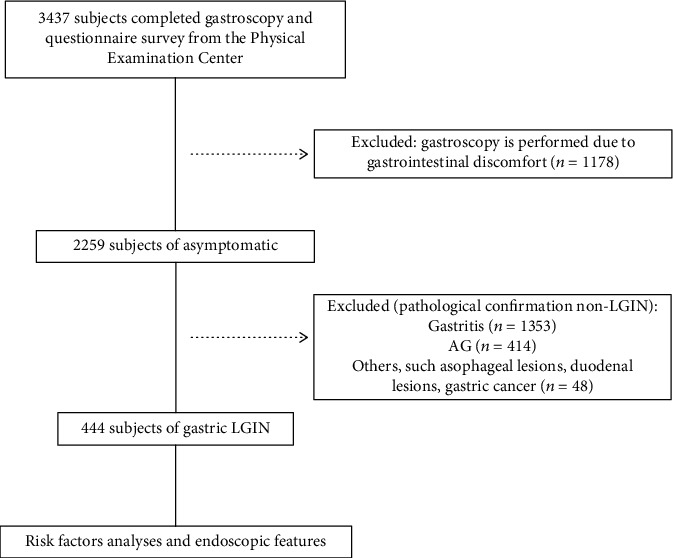
Flow chart for selecting the research population.

In the past four years, we have collected 3437 subjects who underwent gastroscopy from the physical examination center. Among them, 2259 were asymptomatic physical examination and 1178 were physical examination with gastrointestinal symptoms. Among the 2259 cases, the pathological findings of gastric inflammatory (gastritis group), atrophy (AG group, including intestinal metaplasia), LGIN (LGIN group), and other lesions were 1353, 414, 444, and 48, accounting for 59.89%, 18.33%, 19.65%, and 2.12%, respectively. Other lesions included 3 cases of esophageal inflammatory lesions, 4 cases of esophageal LGIN, 1 case of esophageal cancer, 29 cases of gastric polyps, 7 cases of gastric malignancy, and 4 cases of duodenal polypoid lesions. In the symptomatic patients, the gastritis, AG, LGIN, and other groups included 712, 185, 234, and 47, accounting for 60.44%, 15.70%, 19.87%, and 3.99%, respectively. Other lesions included 1 case of esophageal inflammation, 2 cases of esophageal cancer, 39 cases of gastric polyps, 2 cases of gastric malignancy, and 3 cases of duodenal polypoid lesions (Figure 1).

The *H. pylori* infection rate of the overall population was 39.13% (1345/3437), with asymptomatic and symptomatic *H. pylori* infection rates of 35.81% (809/2259) and 45.50% (536/1178), respectively (*P* ≤ 0.001). There was no significant difference between the asymptomatic and symptomatic groups in the three pathological distributions or sex ratio. The rate of *H. pylori* infection with gastrointestinal symptoms was significantly higher than that of the asymptomatic population when the pathological diagnoses were gastritis and atrophy (*P* ≤ 0.001, *P* = 0.003), while there was no significant difference between the two groups in terms of LGIN (Table 1).

### 3.2. Univariate and Multivariate Analyses of LGIN Risk Factors

A total of 12 potential risk factors for LGIN of gastric mucosa were analyzed by univariate analysis. The results showed that age, *H. pylori* infection, history of antibiotic misuse, high-salt diet, spicy and high-fat diet, and poor eating habits were associated with LGIN when compared with the gastritis group (*P* all <0.05) (Table 2).

Multivariate logistic regression analysis further identified age, *H. pylori* infection, antibiotic misuse history, and spicy and high-fat diet as independent risk factors of LGIN. Antibiotic misuse history and *H. pylori* infection showed significant associations with LGIN (odds ratio (OR) = 6.767, 95% confidence interval (CI) 3.873-11.825 and OR = 3.803, 95% CI 3.009-4.808, respectively) (Table 3).

### 3.3. Endoscopic Appearance

We analyzed the endoscopic appearances of 2211 asymptomatic patients and found that the main endoscopic appearances of gastritis were chronic superficial gastritis (45.90%) and erosive gastritis (26.10%). The main appearances of LGIN were erosive gastritis (50.90%) and atrophic mixed erosive gastritis (13.74%) (Table 4).

In accordance with the Paris endoscopic classification of superficial neoplastic lesions, most were described as flat with slight elevation (30.63%) and nodular (16.67%) (Paris IIa) (Table 5) with a high incidence of lesions in the antrum (76.13%) and incisura angularis (16.21%) (Table 6).

### 3.4. Prognosis

A total of 388 cases of LGIN confirmed by pathology were followed up, among which 125 cases (32.22%) were lost over 1 year. Of the 263 cases (67.78%) remaining, endoscopy surveillance was performed in 164 cases, while 99 cases were followed up by telephone. During the average follow-up period of 15.02 (4-41) months of the 164 endoscopic surveillance cases, 82 with LGIN maintained the original pathology (concordant grade group); 81 cases had fewer pathological changes (downgrade group), including 48 cases of atrophic and intestinal metaplasia, 33 cases of gastritis, and 1 case of gastric cancer. That patient was a 65-year-old male, whose firstly endoscopic appearance was erosive lesions of the cardia with *H. pylori* positivity. Eight months later, the possibility of malignancy emerged, soon afterwards endoscopic submucosal dissection (ESD) was performed to remove the lesion. Postoperative pathology showed intramucosal carcinogenesis, which has been followed up regularly. All the subjects who were *H. pylori* positive after endoscopic examination were treated with anti *H. pylori* quadruple therapy. Compared with the concordant grade group, the downgrade group was younger (*P* ≤ 0.001), while there were no differences in *H. pylori* infection or endoscopic classification (Table 7).

## 4. Discussion

Secondary prevention of gastric cancer is an important strategy at this early stage, and the significance of secondary prevention is to screen out subjects at high risk for gastric cancer among the normal population [9]. Previous studies focused on screening patients with explicit gastrointestinal symptoms [10, 11]. However, we know that gastric diseases can be found in the middle and late stages without any symptoms. The incidence of precancerous lesions in asymptomatic population is less reported. This is the first cross-sectional study among asymptomatic subjects, similar to a natural population, therefore avoiding the pitfalls of previous research. More and more people require a gastroscopy exam as part of their health check-up program. Certainly, some people with gastrointestinal symptoms undergo a gastroscopy exam rather than an outpatient visit. Questionnaires are used to separate these groups. LGIN is the last stage of precancerous lesions. The cancer rate of LGIN of the stomach can reach 0.53%-29.6% [12–14], and positive endoscopic treatment is highly effective when necessary [15, 16]. Therefore, timely diagnosis can greatly improve the detection rate of early gastric cancer, leading to reductions in disease incidence, mortality, and associated medical costs.

The incidence of LGIN was 19.65% in the asymptomatic group, which was not significantly different compared with those with gastrointestinal symptoms. A history of antibiotic misuse and *H. pylori* infection were significant risk factors for LGIN among asymptomatic subjects. The most common endoscopic classification of LGIN was erosive gastritis, and its appearance was Paris IIa (flat with slight elevation, located mostly in the antrum). During follow-up, 50% of LGIN maintained the original pathology, 49.4% had fewer pathological changes, and 0.6% progressed to gastric cancer.

In the pooled multivariate analysis, the most significant independent risk factor of LGIN was a history of antibiotic misuse (compared to both the gastritis and atrophic groups). This was considered as taking antibiotics without the guidance of doctors at a frequency ≥ 3 times/year, which is a common phenomenon in China [17]. In our study, the subjects were not previously tested for *H. pylori*, so they did not receive specific antibiotic treatment for that infection. Antibiotics are an important treatment for *H. pylori* infection, but it is not known whether the use of antibiotics alone can cause LGIN of the gastric mucosa. Antibiotics can reportedly cause changes in the gastric microenvironment and lead to carcinogenesis [18]. Antibiotic use seems associated with a slightly elevated breast cancer risk [19], and antibiotic suppression of intestinal microbiota reduces heme-induced lipoperoxidation associated with colon carcinogenesis [20], yet the nature of the association remains elusive.

In pathologically proven cases of gastritis and atrophy, *H. pylori* infection was significantly increased in the symptomatic group, suggesting that *H. pylori* infection may be the cause of gastrointestinal discomfort [21]. Nevertheless there was no significant difference in the LGIN group, which may suggest that we cannot depend symptoms to distinguish disease severity. Consistent with most conclusions, the rate of *H. pylori* infection in the LGIN group was significantly higher than in the gastritis and AG groups. As we know, eradication of *H. pylori* can prevent or reverse the progression of gastric mucosal lesions only in the stage of gastritis [22]. *H. pylori* infection can reduce the scores for atrophy and intestinal metaplasia [23]. A follow-up study of 1755 cases of gastritis in Italy found that *H. pylori*-negative gastritis generally did not progress, and *H. pylori*-positive Operative Link on Gastritis Assessment (OLGA) staging at stage III and IV did not eliminate the risk of the progression to tumorigenesis after *H. pylori* eradication [24]. During follow-up, 59.26% (48/81) of subjects with *H. pylori* infection had downgraded pathological changes after regular treatment, compared to 46.34% (38/82) in the concordant grade group, with no statistical difference between the two groups.

Our study revealed that after age 60, the incidence of LGIN was significantly higher than that in the gastritis group, which was consistent with findings reported by Raftopoulos et al. [25]. We also found that a high-salt diet is a risk factor for gastric LGIN rather than an independent risk factor. Studies showed that a high-salt diet could promote the carcinogenesis of *H. pylori* infection with cytotoxin-associated gene A (*Cag*A *+*), but without a high-salt diet, the *Cag*A*+*mutant does not induce inflammation and low acid levels [26]. Eating a spicy and high-fat diet or poor eating habits are risk factors of LGIN, which is consistent with other studies [27, 28]. These results suggest that consuming a healthy diet and early *H. pylori* eradication are extremely important.

The major endoscopic appearance of LGIN was flat with slight elevation (30.63%) and nodular (16.67%) (Paris IIa), mainly in the antrum, consistent with a 9-year follow-up study of European patients [25]. As Yu et al. [27] pointed out, the proportion of incident proximal LGIN cases increased gradually from 13.6% to 35.3% in 1 year. The single case of gastric cancer found in our follow-up was also located in the gastric cardia, suggesting that we need to strengthen surveillance for lesions in this area.

As new endoscopic treatment technology became available, some LGIN lesions confirmed by biopsy pathology were found to be HGIN, which greatly improved the diagnostic rate of early gastric cancer. Park et al. [14] found that the clinical, morphological, and histological characteristics did not reveal any distinguishable features for progressive dysplasia, and during their median follow-up period of 58 months, 26.9% gastric intraepithelial neoplasia progressed to HGIN or cancer. Zou et al. [16] reported that lesion size ≥2.0 cm and a depressed pattern at the initial endoscopic forceps biopsy were independent risk factors for pathologic upgrade to advanced diseases that required ESD, with an upgraded rate of 25.1%. Yang et al. [15] reported upgrade and downgrade rates of LGIN pathological diagnosis of 48.9% and 12.5%, respectively. A prospective study of 90 cases in Italy [29] showed that 53.3% had spontaneous remission and 31.1% had stable lesions on endoscopic biopsy during a mean of 52-month follow-up (16-206 months). Yamada et al. [30] followed 43 patients for a mean of 6 years (3-18 years); 97% of LGIN remained stable, and 3% progressed to in situ cancer. This suggests that the rate of malignancy is low, and some LGIN can even be reversed. In our study, 49.4% of cases were reversed during the average follow-up of 15months, and only 0.6% progressed to the early gastric cancer, which was consistent with the above studies. We also found that the *H. pylori* infection rate of the reversed group was higher than that of the nonreversed group (59.26% vs. 46.34%). According to Suzuki et al. [31], *H. pylori* infection can produce pathological changes similar to LGIN, and after eradication of *H. pylori* infection, the pathological manifestations of atypical hyperplasia can be improved or even reversed with subsidence of active gastritis. Doorakkers et al. [32] reported that the risk of gastric adenocarcinoma decreased over time after eradication treatment, to levels below that of the corresponding background population. This suggests that it is important to treat *H. pylori* as early as possible. The aforementioned studies had different conclusions probably because they were assessing different populations. Of course, longer follow-up is necessary.

At present, there are many methods for screening those at high-risk of gastric cancer, such as pepsinogen combined with *H. pylori* antibody testing for predicting and screening [33]. OLGA staging for gastritis can also predict the risk of gastric cancer [22], magnetic capsule endoscopy is used to diagnose superficial gastric mucosal lesions [34], and gene screening [35] and vaccine development [36] are all important research methods for improving the detection rate of LGIN.

Of course, this study also has some limitations. It was performed at a single center, and the follow-up time was not sufficient. A history of antibiotic misuse is the predominant risk factor for LGIN, whether it is a widely applicable factor will require additional data. However, to some extent, we systematically analyzed the incidence, risk factors, endoscopic phenotype analysis, and prognosis of LGIN in relatively normal asymptomatic population.

## 5. Conclusions

In summary, we analyzed epidemiological data on the incidence of LGIN of the stomach in people without digestive symptoms. A history of antibiotic misuse and *H. pylori* infection were predominant risk factors of LGIN. Thus, we recommend that people with these risk factors, especially those with several, should consider early screening for gastric cancer.

## Figures and Tables

**Table 1:** General characteristics in the asymptomatic and symptomatic groups.

Variable	Asymptomatic group (*n* = 2211)	Symptomatic group (*n* = 1131)	*P* value
Pathological classification			0.240
Gastritis	1353	712	
AG	414	185	
LGIN	444	234	
Sex (male/female)			
Gastritis	771/582	375/337	0.061
AG	243/171	97/88	0.153
LGIN	269/175	151/83	0.315
Age (*M* ± SD)			
Gastritis	46.39 ± 10.33	45.13 ± 10.46	0.009^∗^
AG	50.37 ± 10.15	49.21 ± 10.43	0.200
LGIN	50.46 ± 10.25	48.97 ± 10.65	0.075
*H. pylori* infection (yes/no)			
Gastritis	387/966	291/421	≤0.001^∗^
AG	138/276	85/100	0.003^∗^
LGIN	273/171	147/87	0.734

^∗^ *P* < 0.05.

**Table 2:** Univariate analysis of common risk factors for LGIN among asymptomatic subjects.

Variable	Gastritis (*n* = 1353)	AG (*n* = 414)	LGIN (*n* = 444)	*P* value	*P* value
				LGIN vs. gastritis	LGIN vs. AG
Sex (male/female)	771/582	243/171	269/175	0.182	0.573
Age (years)	46.39 ± 10.33	50.37 ± 10.15	50.46 ± 10.25	≤0.001^∗^	0.892
≤30	101	11	7	≤0.001^∗^	
31-40	276	61	72	0.053	
41-50	501	138	152	0.288	
51-60	358	129	135	0.106	
61-70	103	64	65	≤0.001^∗^	
>70	14	11	13	0.004^∗^	
BMI (kg/m^2^)	23.12 ± 2.91	23.18 ± 2.85	23.06 ± 2.84	0.693	0.531
*H. pylori* infection (yes/no)	387/966	138/276	273/171	≤0.001^∗^	≤0.001^∗^
NSAIDs (yes/no)	35/1318	14/400	18/426	0.113	0.603
Antibiotic misuse history (yes/no)	23/1330	20/394	43/401	≤0.001^∗^	0.006^∗^
Family history (yes/no)	59/1294	20/394	28/416	0.097	0.347
Smoking history (yes/no)	300/1053	87/327	90/354	0.399	0.788
Alcohol history (yes/no)	336/1017	104/310	106/338	0.684	0.671
High-salt diet (yes/no)	244/1109	72/342	101/343	0.029^∗^	0.051
Spicy and high-fat diet (yes/no)	318/1035	181/233	188/256	≤0.001^∗^	0.684
Poor eating habits (yes/no)	392/961	168/246	162/282	0.003^∗^	0.218

^∗^ *P* < 0.05.

**Table 3:** Multivariate analysis of relevant risk factors for LGIN.

Variables	Β regression coefficient	*P* value	OR (95% CI)
Age	0.027	≤0.001^∗^	1.027 (1.015-1.040)
*H. pylori* infection	1.336	≤0.001^∗^	3.803 (3.009-4.808)
Antibiotic misuse history	1.912	≤0.001^∗^	6.767 (3.873-11.825)
High-salt diet	0.154	0.297	1.166 (0.874-1.556)
Spicy and high-fat diet	0.661	≤0.001^∗^	1.936 (1.498-2.502)
Poor eating habits	0.217	0.092	1.243 (0.965-1.601)

^∗^ *P* < 0.05.

**Table 4:** Endoscopic classifications of gastritis, AG, and LGIN.

Variable	Gastritis (*n* = 1353)	AG (*n* = 414)	LGIN (*n* = 444)	*P* value	*P* value
				LGIN vs. gastritis	LGIN vs. AG
Chronic superficial gastritis	621 (45.90%)	67 (16.18%)	54 (12.16%)	≤0.001^∗^	0.091
Pangastritis	208 (15.37%)	31 (7.49%)	36 (8.11%)	≤0.001^∗^	0.735
Chronic atrophic gastritis	89 (6.58%)	59 (14.25%)	47 (10.59%)	0.006^∗^	0.103
Erosive gastritis	353 (26.10%)	180 (43.48%)	226 (50.90%)	≤0.001^∗^	0.030^∗^
Atrophic mixed erosive gastritis	51 (3.77%)	69 (16.67%)	61(13.74%)	≤0.001^∗^	0.232
Gastric ulcer	31 (2.29%)	8 (1.93%)	20 (4.50%)	0.015^∗^	0.034^∗^

^∗^ *P* < 0.05.

**Table 5:** Endoscopic appearances of LGIN in accordance with the Paris classification.

Endoscopic appearance	*n*	%
Polypoid		
Pedunculated (Paris Ip)	2	0.45
Protruded (Paris Is)	5	1.13
Nonpolypoid		
Slightly elevated (Paris IIa)		
Irregular mucosa	73	16.44
Nodular	74	16.67
Flat with slight elevation	136	30.63
Flat (Paris IIb)		
Erythema	40	9.00
Flat	23	5.18
Gastritis	24	5.41
Inconspicuous	12	2.70
Slightly depressed (Paris IIc)		
Erosions	32	7.21
Flat depressed	1	0.23
Excavated (Paris 0-III)		
Ulcerated	22	4.95
Total	444	100

**Table 6:** LGIN lesion locations.

Location	*n*	%
Antrum	338	76.13
Funds	18	4.05
Incisura angularis	72	16.21
Pylorus	7	1.58
Cardia	9	2.03

**Table 7:** The general and endoscopic characteristics of LGIN prognosis.

	Concordant group (*n* = 82)	Downgrade group (*n* = 81)	*P* value
Sex (male/female)	53/29	49/32	0.585
Age (years)	53.43 ± 10.96	47.91 ± 9.45	≤0.001^∗^
*H. pylori* infection (yes/no)	38/44	48/33	0.099
Antibiotic misuse history	7/75	3/78	0.199
Spicy and high-fat diet	35/47	34/47	0.927
Endoscopic classification			0.566
Chronic superficial gastritis	12	7	
Pangastritis	4	4	
Chronic atrophic gastritis	5	9	
Erosive gastritis	41	44	
Atrophic mixed erosive gastritis	16	11	
Gastric ulcer	4	6	
Endoscopic classification			
Polypoid			
Pedunculated (Paris Ip)	0	0	
Protruded (Paris Is)	0	2	
Nonpolypoid			
Slightly elevated (Paris IIa)			
Irregular mucosa	9	20	
Nodular	16	13	
Flat with slight elevation	28	18	
Flat (Paris IIb)			
Erythema	7	5	
Flat	2	6	
Gastritis	7	3	
Inconspicuous	3	2	
Slightly depressed (Paris IIc)			
Erosions	6	5	
Flat depressed	0	0	
Excavated (Paris 0-III)			
Ulcerated	4	7	

^∗^ *P* < 0.05.

## Data Availability

The testing methods and experimental data used to support the findings of this study are included within the article and the supplementary information file.

## Supplementary Materials

Supplementary Materials Table S1: questionnaire survey of gastroscopy for physical examination subjects.
