# Supplementary material for: Analysis of Risk Factors of Gastric Low-Grade Intraepithelial Neoplasia in Asymptomatic Subjects Undergoing Physical Examination
Source: Gastroenterol Res Pract. 2020 Mar 3;2020:7907195. doi: 10.1155/2020/7907195 (PMC7072095; doi:10.1155/2020/7907195)
Supplement: Supplementary Materials — Table S1: questionnaire survey of gastroscopy for physical examination subjects. [file 7907195.f1.pdf]

Table S1 Questionnaire survey of gastroscopy for physical examination subjects

1. Baseline information

Name: ;Age (years); Gender: male/female ;

Height (cm): ;Weight (kg): ;Body mass index (BMI,kg/m^2): ;

Home address:

Contact number :

2. The purpose of gastroscopy

☐ Physical examination, no gastrointestinal discomfort.

☐ Gastrointestinal discomfort or slight physical discomfort Symptoms of gastrointestinal discomfort (one or more)

☐ abdominal pain, ☐abdominal distention, ☐acid reflux, ☐belching, ☐early satiety,☐postprandial discomfort, ☐heartburn, ☐diarrhea , ☐hematemesis, ☐anorexia, ☐dysphagia, ,☐ poststernal discomfort, ☐nausea,☐ vomiting, ☐others

3. Personal history

1) NSAIDs history:Yes/No

Note:NSAIDs history(Nonsteroidal Antiinflammatory Drugs)means the long-term use due to cardiovascular , rheumatoid diseases or other painful diseases with duration >6 months.

2)Misuse antibiotic history:Yes/No

Note:misuse of antibitic history means taking antibiotic without the guidance of doctors ≥3 times/yr

3)Family history of gastric cancer:Yes/No

Note:family history of gastric cancer means the history of first-degree relatives of gastric cancer

4)Smoking history:Yes/No

Note:smoking history means average daily cigarette consumption over 3 packs of cigarettes/wk with duration ＞6 months.

5) Alochol history: Yes/No

Note: alochol history means average daily alcohol consumption ＞100ml over twice per week with duratuion ＞1yr.

6) High salt diet: Yes/No

Note: high salt diet means average daily salt consumption is over 6 g daily.

7) Spicy and high fat diet: Yes/No

Note: spicy and high fat diet means year-long daily consumption of spicy and high fat diet ≥3 times/wk

8) Poor eating habit:

Note: poor eating habit means irregular dietary habits.eg,no breakfast consumption, picky eaters,vegetarion or meat only.
